# Supplementary material for: 3D-printed sheet jet for stable megahertz liquid sample delivery at X-ray free-electron lasers
Source: IUCrJ. 2023 Sep 18;10(Pt 6):662–70. doi: 10.1107/S2052252523007972 (PMC10619454; doi:10.1107/S2052252523007972)
Supplement: Supplementary file 3 [file m-10-00662-sup3.pdf]

IUCrJ

Volume 10 (2023)

Supporting information for article:

3D-printed sheet jet for stable megahertz liquid sample delivery at X-ray free-electron lasers

Patrick E. Konold, Tong You, Johan Bielecki, Joana Valerio, Marco Kloos, Daniel Westphal, Alfredo Bellisario, Tej Varma Yenupuri, August Wolter, Jayanath C. P. Koliyadu, Faisal H.M. Koua, Romain Letrun, Adam Round, Tokushi Sato, Petra Mészáros, Leonardo Monrroy, Jennifer Mutisya, Szabolcs Bódizs, Taru Larkiala, Amke Nimmrich, Roberto Alvarez, Patrick Adams, Richard Bean, Tomas Ekeberg, Richard A. Kirian, Andrew V. Martin, Sebastian Westenhoff and Filipe R. N. C. Maia

Supporting Information

3D-Printed Sheet Jet for Stable Megahertz Liquid Sample Delivery at X-ray Free Electron Lasers

Patrick E. Konold *et al.*

Main chamber vacuum levels

Without jet: 8e-6 mbar
With GDVN: 2.9e-5 mbar
With Sheet jet: 4.3e-5 mbar

3D Printing parameters

Voxel height: 8.87 µm
Voxel width: 0.735 µm
Laser power: 85 mW
Infill speed: 600 mm/s
XY stage speed: 1 mm/s
Z stage speed: 0.5 mm/s
Block height: 60 µm
Field of view size: 500 µm
Field of view overlap: 10 µm

Special care was given to avoid stitching lines falling within the channel regions. As such, a complete channel would be printed within the 60 µm block for a single field of view.

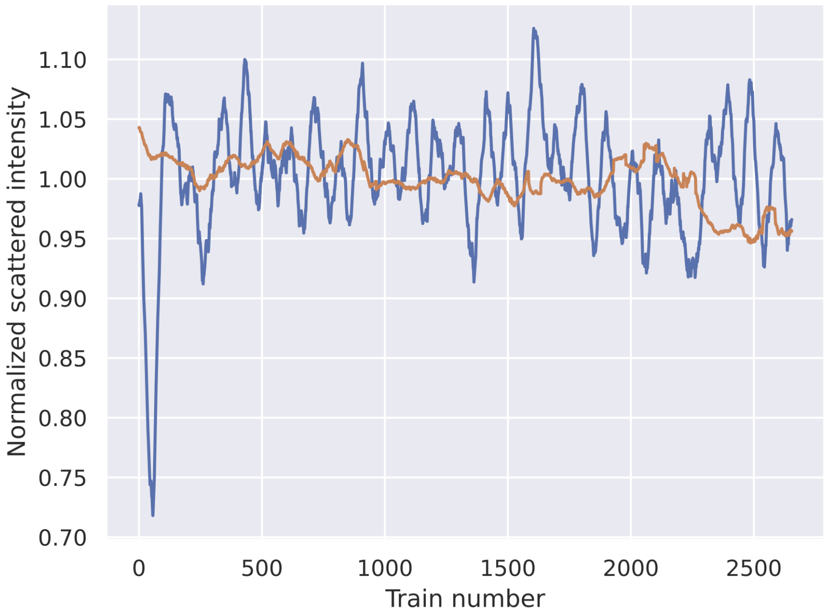

Figure S1. The integrated AGIPD detector response normalized by incoming X-ray intensity binned in 50 train intervals over a representative 5 minute measurement window for the liquid sheet jet and GDVN

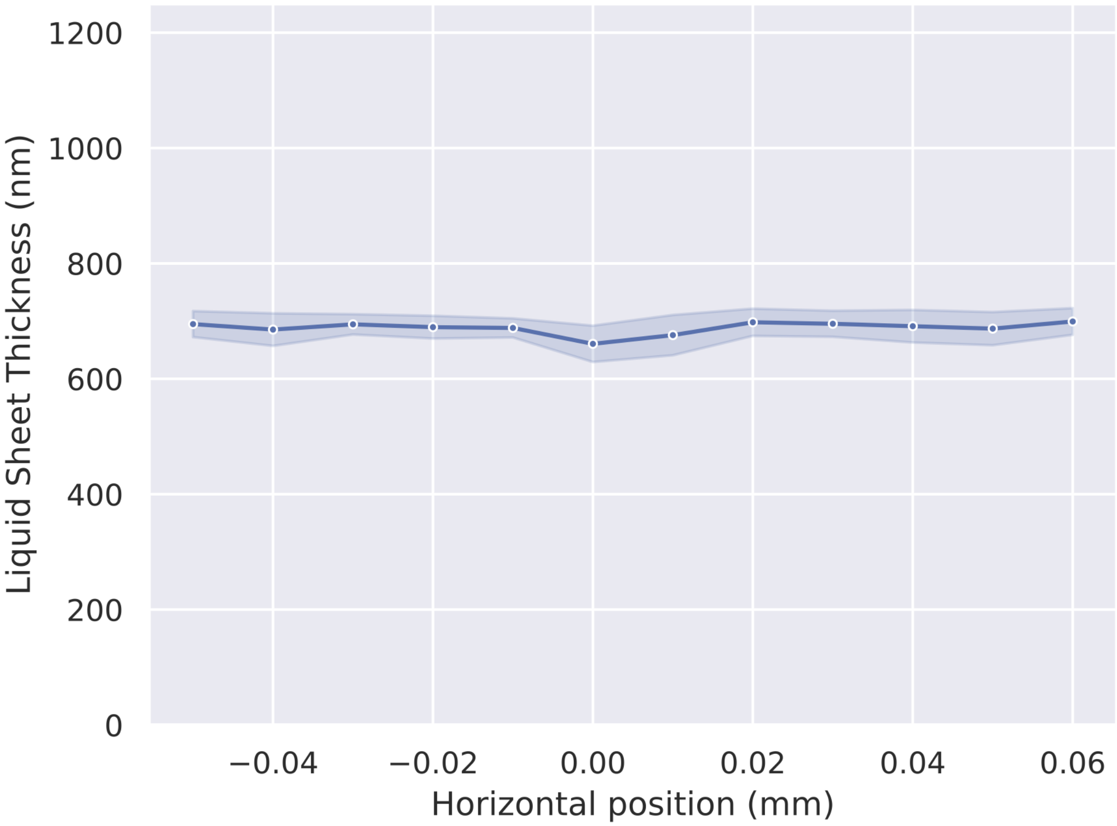

Figure S2. Thickness values extracted from scanning horizontally across the primary liquid sheet region at a distance of 185 μm from the nozzle tip.

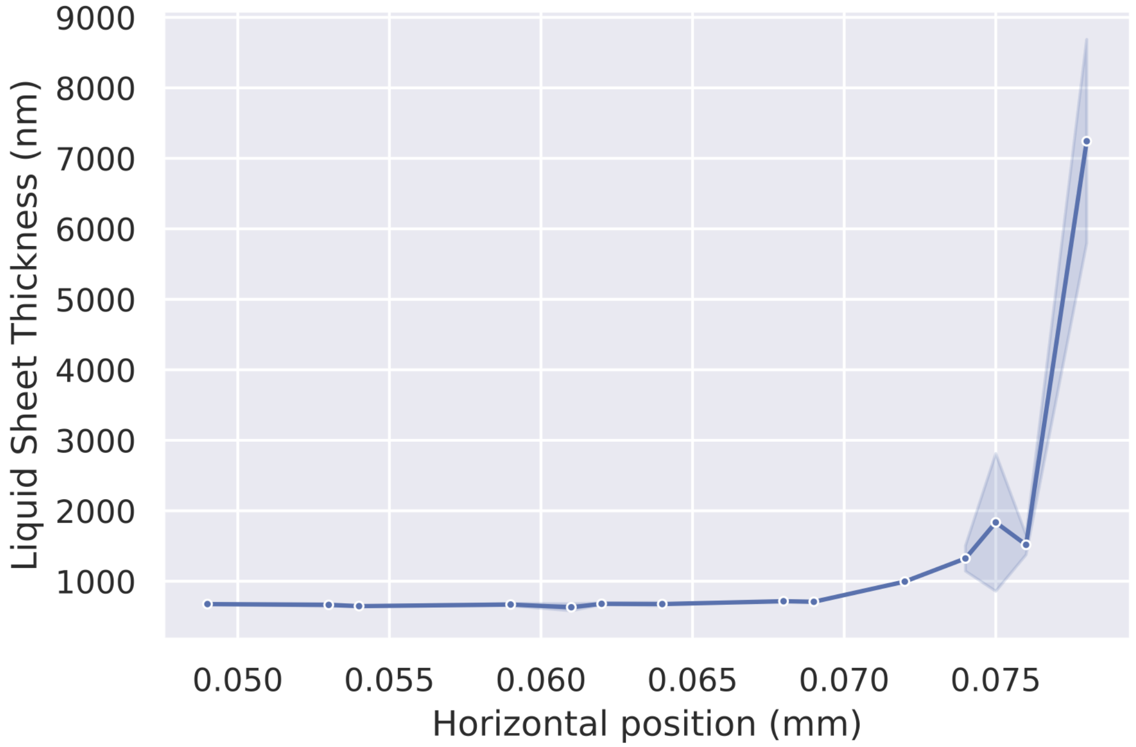

Figure S3. Same horizontal scan as in **Fig. S2**, but here we show the part of the scan where the X-rays hit the rim of the liquid sheet. The sheet has constant thickness until very close to the rim. The increased thickness close to the rim may be due to the tails of the X-ray beam clipping the rim.

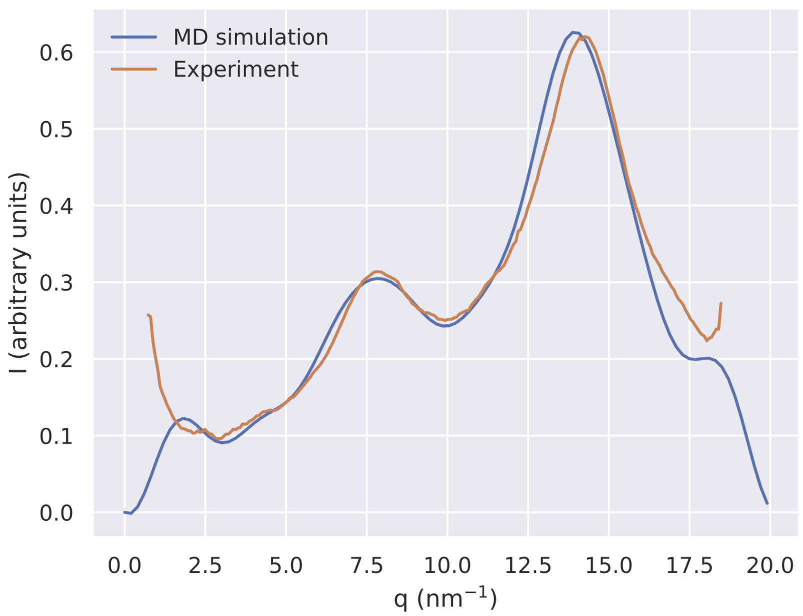

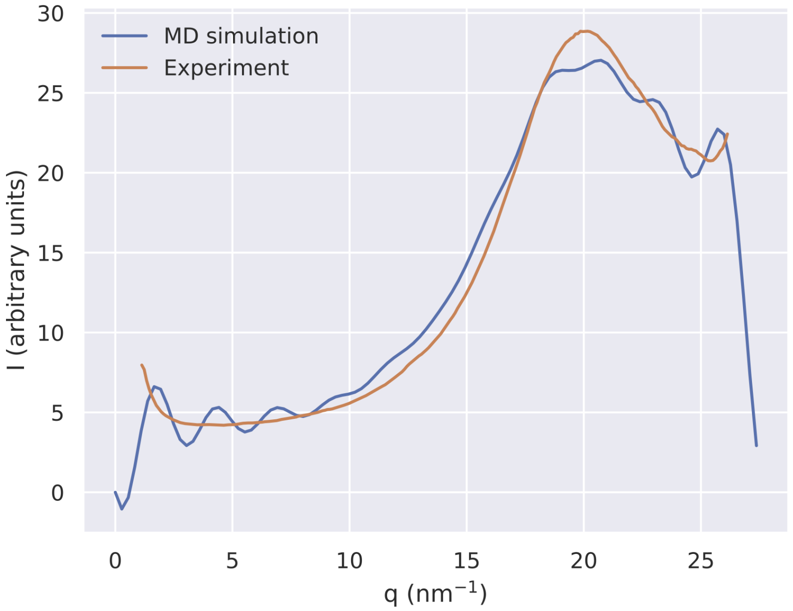

Figure S4. Scattering curves derived from molecular dynamics simulations for isopropanol (above) delivered by the liquid sheet and for water (below) delivered by a GDVN.

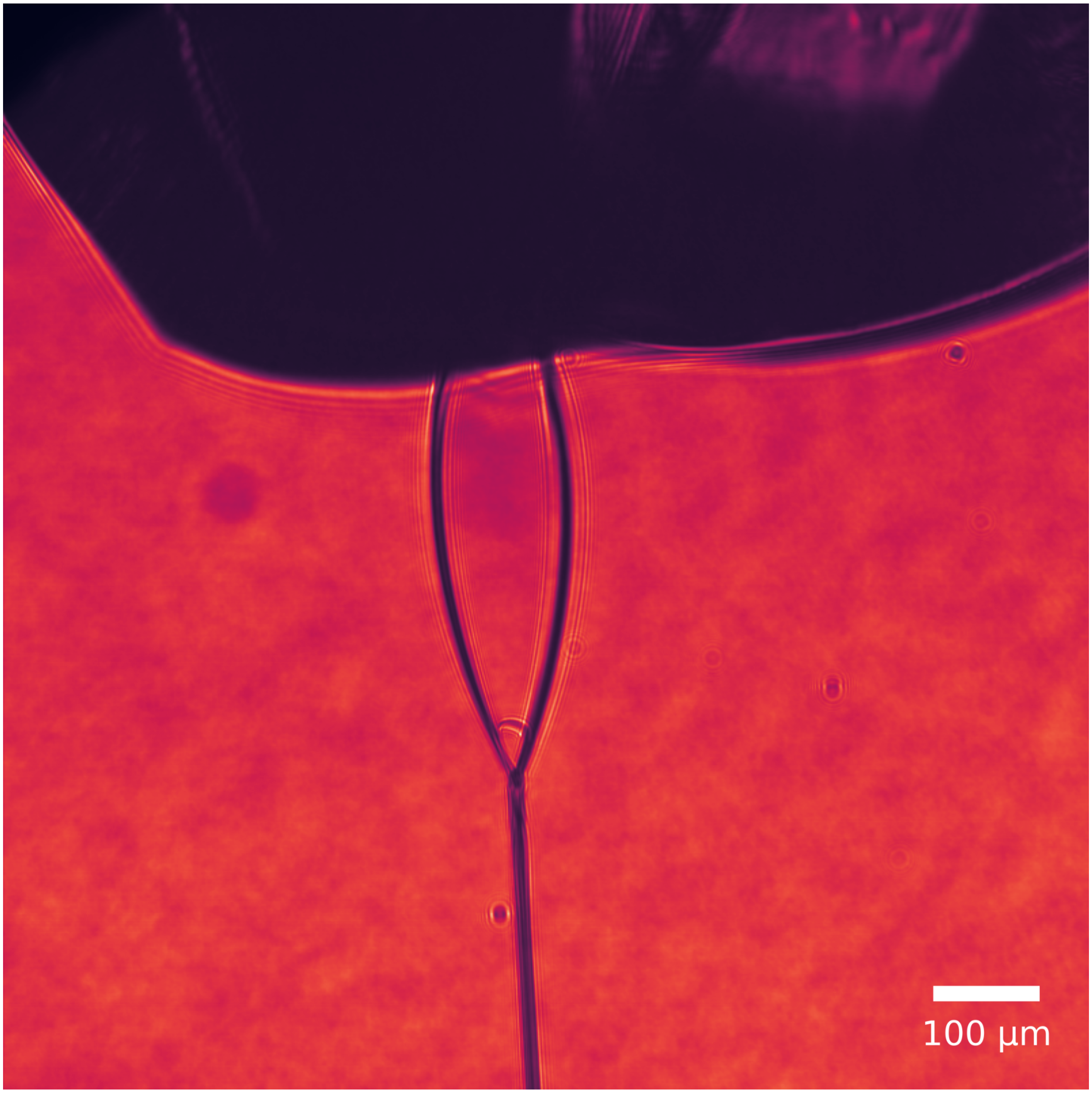

Figure S5. Side view microscope image of the liquid sheet when the X-rays intersect the sheet 350 µm below the nozzle, corresponding to the thinnest part of the sheet (65 nm) as shown in Fig. 2b. The width of the sheet at this position when projected onto the microscope, which is at a 45 degree angle, is ~32 µm. This gives a real sheet width of 45 µm.

Movie S1. run 194

Movie S2. run 186
